# Changes in drug demand when a pandemic coincides with other outbreaks in a war zone country: a cross-sectional pilot study

**DOI:** 10.1186/s40545-022-00487-z

**Published:** 2022-11-22

**Authors:** Ebtesam A. Saleh, Randa N. Haddadin, Bassam Saleh, Eman Elayeh

**Affiliations:** 1 Department of Pharmacology & Toxicology, School of Pharmacy, University of Aden, Aden, Yemen; 2 Department of Pharmaceutics and Pharmaceutical Technology, School of Pharmacy, The University of Jordan, Amman, Jordan; 3 Medical and Health Unit, International Organization of Migration (IOM), Aden, Yemen; 4 Department of Biopharmaceutics and Clinical Pharmacy, School of Pharmacy, The University of Jordan, Amman, Jordan

**Keywords:** COVID-19, Pharmacy, Self-medication, Outbreaks, Yemen

## Abstract

**Background:**

Yemen's health system has severely deteriorated due to the ongoing civil war accompanied by the COVID-19 pandemic which coincided with other outbreaks of endemic infections. Many health institutions closed due to insufficient equipment and supplies. Consequently, pharmacists became the available healthcare provider on the frontlines. This study aimed to evaluate the pattern of demand for prescription and nonprescription drugs during the pandemic based on the pharmacist's perspective in Yemen, a conflict zone country.

**Methods:**

An online survey was developed, validated, and distributed among pharmacists. The questionnaire was composed of two sections: (1) demographic characteristics of the participants and (2) changes in the demand for different drug categories. Chi-square test and Fisher's exact test were used to find statistical associations.

**Results:**

Responses (330) were received from pharmacists working in 12 out of 22 governorates in Yemen. During the pandemic, there was an increase in the demand for prescription drugs such as antibiotics, antimalarials, and sedatives (95%, 90%, and 71%, respectively) and an increase in the demand for nonprescription drugs such as vitamins (93%) and paracetamol (> 90%). Some of these drugs could have serious side effects if taken without medical advice, and others could result in severe effects if taken concomitantly. In addition, there was an increase in the demand for prescription drugs without a prescription, which was reported by 50% of the participants. No statistical difference was seen between the frontline districts and major cities in terms of requesting these drugs without a prescription. On the other hand, most participants (75.2%) did not attend any training or workshop during the last 6 months of conducting this survey.

**Conclusion:**

COVID-19 has increased the demand for many prescription and nonprescription drugs, where the irrational use of these drugs may lead to devastating health effects. In war zone areas hit by a pandemic, policymakers and public health organizations should focus on training and educating pharmacists as important health care and medicine providers for the public.

**Supplementary Information:**

The online version contains supplementary material available at 10.1186/s40545-022-00487-z.

## Background

In the year 2020, an outbreak of a novel coronavirus spread in 219 countries, and by the end of that year, it affected more than 79 million people and claimed the lives of over 1.7 million [1]. The World Health Organization (WHO) declared the disease a pandemic under the name Coronavirus disease 2019 (COVID-19) [2]. Yemen, a Middle Eastern country shattered by a civil war since 2015, was not away from the pandemic. On April 10, 2020, the first confirmed cases were announced, and in January 2021, the confirmed cases reached 2119 cases and 615 deaths [3]. However, the actual reported cases of COVID-19 and deaths were inaccurate, and the actual escalation of cases has been impossible to be determined due to insufficient testing capabilities and lack of transparency from the government and the rebels [4]. Earlier in 2020, WHO announced that the health system in Yemen was almost collapsed due to the ongoing civil war, which was accompanied by seasonal outbreaks such as cholera, dengue fever, malaria, chikungunya, and diphtheria, where only 45% of the healthcare facilities were fully functioning [5, 6]. Therefore, the emergence of COVID-19 in Yemen was described as “a crisis within crises” [7]. It aggravated the humanitarian crisis and was highly devastating to the healthcare system, where within 3 months, about 97 medical staff died due to coronavirus [8].

The healthcare facilities were incapable of dealing with COVID-19 patients due to shortages in the necessary equipment and isolation rooms. In addition, a high incidence of infections and mortality were reported among health staff due to COVID-19 [8]. All these conditions pushed the healthcare staff to leave these facilities [9]. Under these circumstances, the healthcare system was reshaped in Yemen, where pharmacists became the frontline dealing with increasing numbers of patients with suspected symptoms of COVID-19 or other ailments.

In general, during the COVID-19 pandemic, the role of pharmacists significantly changed in different countries. For instance, the International Pharmaceutical Federation and the American Pharmacist Association have both issued guidelines to prepare and strengthen pharmacists as frontline healthcare workers during the pandemic [10, 11]. In Australia, pharmacists dispensed controlled drugs using prescriptions that were electronically received via e-mail or fax [12]. In Yemen, the health care system and the pharmaceutical sector were severely deteriorated due to the civil war since 2015 [13, 14]. The legislation is outdated or not enforced which was reflected on pharmacy practice. Several studies have reported an increase in medication safety problems, ease of access to prescription drugs without prescription [15], medication dispensing errors, counterfeit drugs, and pharmacists' outdated education [13, 16]. In addition, irrational self-medication with antibiotics among adults and children, and drug abuse of sedatives and opioid analgesics have been reported [17, 18]. COVID-19 has worsened self-medication behavior, where this behavior was reported among people who had never self-medicated before the pandemic [19]. These people will procure their medications from the pharmacies. Accordingly, under the pressure posed by the pandemic on the health system accompanied by seasonal epidemics and in a war-torn country like Yemen, the pharmacist's role became critical in pharmaceutical care, rational drug dispensing, and patients' education [20]. Therefore, since the world is expected to face many other pandemics in the future, and other countries could be in the same situation of Yemen (a war zone country suffering from local outbreaks), it is crucial for international organizations to study and understand the lessons gained from Yemen. It is important to investigate the changes in the demand for drugs, study the factors associated with these changes, explain the reasons behind these changes, and analyze the impact of the changes on people’s health. This would help policymakers to make proper decisions related to drug supplies, regulations related to drug prescription and dispensing, training and educating pharmacists, and any need to launch awareness campaigns targeting the general public.

## Methods

### The study's aim, design, and data collection

The study aimed to evaluate the changes in the demand for selected drugs, whether prescription or nonprescription, during COVID-19 in Yemen based on the pharmacist's perspective, and to investigate factors associated with changes in drug demand in Yemen during the study period. An online survey was conducted for 3 months (March–May) in 2020, targeting Yemeni pharmacists working in pharmacies (community and hospital pharmacies) at the time of the study during the COVID-19 pandemic. An online survey was preferred over distributing hard copies due to the difficulty in moving between war zone districts in addition to the risks posed by the pandemic.

The survey was developed after an extensive literature review [18, 21, 22]. Initially, a group of hospital and community pharmacists were contacted to suggest a list of the most common drugs and drug groups to which they have noticed an increase in their demand during COVID-19 and to provide any other comments they have observed related to medication demand. The authors were able to secure responses from 10 pharmacists who prepared lists of drugs and drug groups to which they have observed changes in their demand. The list was refined and included in the survey. The survey was first designed in English (Additional file 1), and then translated into Arabic language, where the medication names were kept in Arabic and English for participants’ convenience. The initial drafts of the English and Arabic versions were circulated among the research team to check for the clarity and readability of the questions. The translation was checked by three academicians who have a pharmacy degree and are native Arabic speakers and finished their higher degrees from western universities which teach in English. The two versions of the survey were face and content validated by a clinician, a clinical pharmacist, and two academicians who are fluent in both languages to ensure the suitability of the questions for Yemeni pharmacists. The Arabic version was pretested on a sample of 18 community pharmacists. This sample was excluded from the pivotal study. The questionnaire was composed of two sections: 1) demographic and personal data, which was concerned with the participant training and workshops attended in the last 6 months and the source of information about COVID-19, and 2) changes in the demand for different drug categories from the pharmacist perspective and whether prescriptions for prescription-only drugs were available or not.

The questionnaire was then developed as a Google form and disseminated to participants through social media such as WhatsApp and Facebook. The survey was posted on different pharmacists’ groups on these platforms.

The study's objectives were stated on the home webpage of the questionnaire, and the participants had the choice to accept (to consent to participate) or refuse to fill out the questionnaire.

### Study population and sampling

In Yemen, the Ministry of Health and the Medical Council include both pharmacy university graduates (BSc degrees and higher) and pharmacy diploma graduates within pharmacy professionals [14]. In a study by Al-Worafi (2014), only 10% of pharmacists working in pharmacies and drug stores have graduated from government-approved colleges. The rest (90%) are either non-pharmacists or graduated from unrecognized institutes. Therefore, in this study, the study population consisted of pharmacists (university graduates), pharmacy technicians (diploma holders), and undergraduate pharmacy students who were dealing with patients in community and hospital pharmacies in different governorates in Yemen during the pandemic of COVID-19. In the context of this study, the term “pharmacist” refers to anyone in these three categories.

The inclusion criteria included anyone who dispenses medicine in a community or hospital pharmacy and who has a university degree in pharmacy (BSc and higher) or diploma or is an undergraduate pharmacy student. Any respondent who did not fulfill these criteria or did not complete the whole questionnaire was excluded from the study.

As a result of the ongoing war in Yemen since a decade ago, accurate records of the official pharmacies or pharmacists registered are scarce. In addition, many difficulties were encountered in recruiting participants for this study due to weak or no internet connection and frequent electricity outages as a result of the war. This situation was aggravated by the pandemic. Therefore, we reviewed the recently published studies conducted among Yemeni pharmacists to determine the appropriate sample size. In the reviewed studies, sample sizes ranging from 153 to 450 were used in similar published studies [15, 23, 24]. Accordingly, a convenient sample of 330 pharmacists was found suitable.

### Statistical analysis

Statistical analysis was performed using SPSS (Statistical Package for Social Sciences) version 20.0 (SPSS Inc., Chicago, IL). Descriptive statistics were used to describe the demographic characteristics of participants. Categorical variables were presented as valid percentages while continuous variables were presented as mean with standard deviation. The study’s variables were the change in the demand for drugs which was measured by counting frequencies and factors affecting the changes in drug demand. Bivariate analyses to find associations between categorical variables (change in demand and pharmacy type or district category) were carried out using the Chi-square test or Fisher’s exact test. All hypothesis testing was two-sided. A p-value of < 0.05 was considered significant.

## Results

### Demographic data

Three hundred and forty-five responses were received from the participants. Only 330 participants completed the entire questionnaire correctly. Most of the participants were males (60.6%), and three-quarters (74.8%) had graduated from governmental universities in Yemen, where 60.3% of the participants had a bachelor's degree. Almost half the participants (50.6%) had a work experience of more than 5 years.

Due to the civil war situation, we categorized the districts according to the conflict dynamics in Yemen into unstable districts, those which have occasional armed conflicts and missile attacks and these include the major cities such as Aden, Lahij, Sana'a, Hadramout, and Al-Hudaida, and in front-line districts such as Al-Dhale, Abyan, Marib, Shabowa, and Taiz.

Aden and Sana'a districts have the highest proportions of participants (27.3% and 21.5%, respectively). Sana’a was the capital of the country until the beginning of the civil war in 2015. It has a population of around two million. The port city of Aden has a population of around 800,000, and it has been the economic capital since 1990 [25, 26]. The demographic characteristics of the participants and the location of the pharmacies are summarized in Table 1.

**Table 1:** Characteristics of participants in the study

Variable	% (n)
Gender	
Males	60.6 (200)
Females	39.4 (130)
Academic degree	
Undergraduate student	11.8 (39)
Diploma	26.4 (87)
Bachelor's degree	60.3 (199)
Postgraduate (MSc. /PhD)	1.5 (5)
Others	0
University of graduation	
Governmental school in Yemen	74.8 (247)
Private school in Yemen	24.5 (81)
In Levant countries	0.3 (1)
In Western Europe or North America	0.3 (1)
Russia	None
District	
Unstable districts "occasional armed conflicts and missile attacks"	
Aden	27.3 (90)
Sana'a	21.5 (71)
Hadramout	16.1 (53)
Lahij	5.5 (18)
Al-Hudaidah	5.2 (17)
Front-line districts	
Taiz	5.8 (19)
Shabwoa	4.8 (16)
Al-Dhale'e	5.8 (19)
Abyan	7.3 (24)
Others	0.9 (3)
Job description	
Hospital pharmacy	21.2 (70)
Community pharmacy	57.9 (191)
Pharmacy owner	11.2 (37)
Trainee in pharmacy	9.7 (32)
Years of experience	
Still student	9.7 (32)
< 1 year	2.1 (970)
1–5 years	36.7 (121)
> 5 years	50.6 (170)
Training/workshops during the last 6 months	
0	75.2 (248)
1	12.4 (41)
2	5.2 (17)
3	3.3 (11)
4	0.9 (3)
5	3.0 (10)
> 5	0
Update on COVID-19	
Yes	93.9 (310)
No	6.1 (20)

### Participants’ information regarding COVID-19

**Fig. 1:**
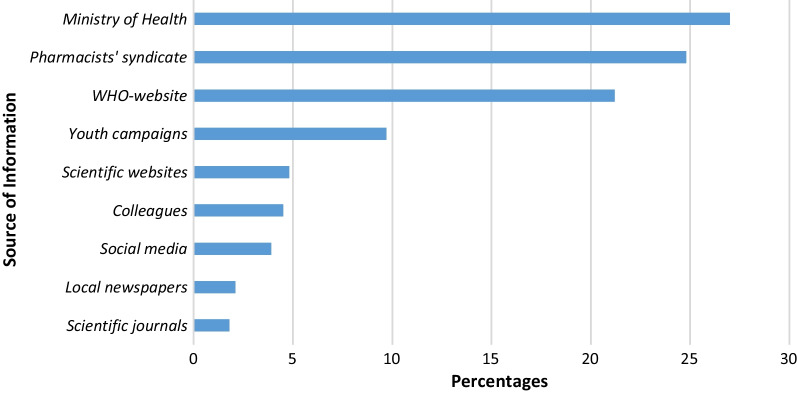
Sources of participant information about COVID-19 and its management

The vast majority of the participants (93.9%) confirmed that they were updated regarding COVID-19 (Table 1). The Ministry of Health, Pharmacists' syndicate, and WHO website were the most common sources of information used by the participants, as shown in Fig. 1.

## Patterns of drug demand

The perceived increase in the demand for different drug classes is summarized in Table 2.

**Table 2:** The increase in drug demand during COVID-19 in Yemen as perceived by participants (*N* = 330)

Drug demand by pharmacological category	% (*n*)
1. Antibiotics	
No, the demand did not change	4.8 (16)
Yes, the demand increased	95.2 (314)
- Macrolides	90.6 (299)
- Parenteral cephalosporins	78.5 (259)
- Penicillin	60.6 (200)
- Oral cephalosporins	58.8 (194)
- Fluoroquinolone	0
- Aminoglycosides	0
2. Analgesics and antipyretics	
No, the demand did not change	1.2 (4)
Yes, the demand increased	98.8 (326)
- Oral paracetamol	90.9 (300)
- Parenteral paracetamol	90.3 (298)
- Paracetamol–codeine combinations	74.8 (247)
- Parenteral diclofenac	44.5 (147)
- Oral diclofenac	26.1 (86)
- Oral ibuprofen	24.2 (80)
- Oral meloxicam	17.0 (56)
- Parenteral meloxicam	7.3 (24)
- Oral tramadol	10.6 (35)
- Parenteral tramadol	4.8 (16)
3. Antithrombotic	
No, the demand did not change	33.0 (109)
Yes, the demand increased	67.0 (221)
- Aspirin	54.8 (181)
- Enoxaparin	46.1 (152)
- Clopidogrel	9.4 (31)
- Heparin	5.2 (17)
- Warfarin	1.8 (6)
4. Anxiolytics and sedatives	
No, the drug demand was unchanged	29.4 (97)
Yes, the demand increased	70.6 (233)
- Benzodiazepines	38.8 (128)
- Barbiturates	4.2 (14)
- Opioids	0.6 (2)
- Antidepressants	5.2 (17)
- Pregabalin	27.6 (91)
5. Antivirals (e.g., oseltamivir, acyclovir, amantadine)	
No, the demand did not change	70.7 (234)
Yes, the demand increased	29.3 (97)
6. Antimalarials (e.g., hydroxychloroquine, chloroquine, artemether, etc.)	
No, the demand did not change	38.7 (128)
Yes, the demand increased	61.3 (203)
7. Ondansetron (anti-emetic drug) demanded with one of the following medicines: antimalarial, macrolides, fluoroquinolone	
Yes	74.8 (247)
No	13.6 (45)
Vitamins and supplements	
Yes	93.0 (307)
No	7.0 (23)

Analgesics and antipyretics reported the highest increase in demand (98.8%) followed by, antibiotics (95.2%), vitamins and supplements (93%), anxiolytics and sedatives (70.6%), antithrombotics (67%) and antimalarials (61.3%). In addition, an increase in the demand for ondansetron (a prescription anti-emetic drug) together with antimalarials or macrolides/fluoroquinolone was reported by the participants (74.8%).

Among analgesics, both oral and parenteral paracetamol recorded the highest increase in demanded drugs (90.9% and 90.3%, respectively), followed by paracetamol–codeine combinations and parenteral diclofenac (74.8% and 44.5%, respectively; Table2).

The distribution of the increase in demand for antibiotics is shown in Table 2. The highest increase in demand is seen for macrolides and parenteral cephalosporins, followed by penicillin and oral cephalosporins. The participants reported no increase in the demand for fluoroquinolones or aminoglycosides during the study period (Table 2). Among the antithrombotic drugs, aspirin scored the highest increase in demand (54.8 %), followed by enoxaparin (46.1 %).

**Fig. 2:**
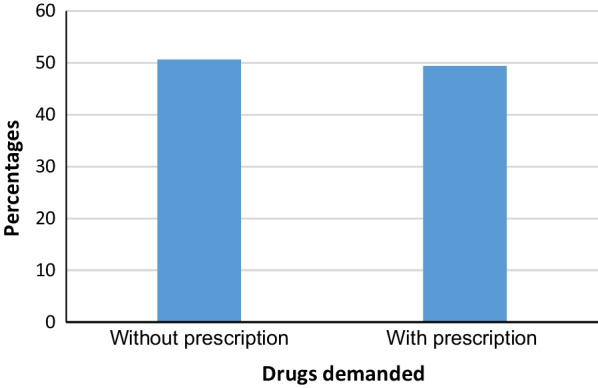
The proportion of prescription drugs demanded with or without providing a prescription as perceived by the participants (*N* = 330)

As demonstrated in Fig. 2, almost half of the participants (50.6%) indicated that their customers did not provide any valid prescription for prescription drugs.

Table 3 shows the association of drugs demanded with the pharmacy type (community/hospital pharmacy) and the pharmacy's location in terms of districts. A significant association between the increase in the demand for vitamins and supplements from community pharmacies was seen (Pearson Chi-square test, *p*-value = 0.012). Besides that, the participants noticed that ondansetron was usually demanded concomitantly with antimalarials or macrolides in unstable districts more than in front-line districts (Pearson Chi-square test, *p*-value= 0.026).

**Table 3:** Factors that may affect drug demand patterns during COVID-19 in Yemen as perceived by the participants (*N* = 330)

Drug categories	Pharmacy parameters cross-tabulated with the drug demand patterns			
	Pharmacy type		District category	
	Community	Hospital	Unstable districts	Front-line districts
1. Antibiotics				
Increased	95.6 (218)	94.3 (66)	96.0 (240)	92.5 (74)
Unchanged	4.4 (10)	5.7 (4)	4.0 (10)	7.5 (6)
*P* value	0.746*		0.232*	
Pearson Chi-square value	0.211		1.609	
2. Antivirals				
Increased	25.9 (59)	35.7 (25)	30.8 (77)	25.0 (20)
Unchanged	74.1 (169)	64.3 (45)	69.2 (173)	75.0 (60)
*P* value	0.110*		0.526*	
Pearson Chi-square value	2.560		1.40	
3. Antimalarials				
Increased	64.5 (147)	52.9 (37)	60.0 (150)	65.0 (52)
Unchanged	35.8 (81)	47.1 (33)	40.0 (100)	35.0 (28)
*P* value	0.08*		0.652^#^	
Pearson Chi-square value	3.060		1.271	
4. Anxiolytics and sedatives				
Increased	70.2 (160)	70.0 (49)	71.2 (178)	68.7 (55)
Unchanged	29.8 (68)	30.0 (21)	28.8 (72)	31.3 (25)
*P* value	0.978^#^		0.675^#^	
Pearson Chi-square value	0.001		0.175	
5. Antithrombotic				
Increased	66.2 (151)	64.3 (45)	67.2 (168)	66.3 (53)
Unchanged	33.8 (77)	35.7 (25)	32.8 (82)	33.7 (27)
*P* value	0.764^#^		0.875^#^	
Pearson Chi-square value	0.090		0.025	
6. Analgesics and antipyretics				
Increased	99.1 (226)	98.6 (69)	99.2 (248)	97.5 (78)
Unchanged	0.9 (1)	1.4 (2)	0.8 (2)	2.5 (2)
*P* value	0.554*		0.248*	
Pearson Chi-square value	0.163		1.463	
7. Vitamins and supplements				
Increased	94.7 (216)	85.7 (60)	94.0 (235)	90.0 (72)
Unchanged	5.3 (12)	14.3 (10)	6.0 (15)	10.0 (8)
*P* value	0.012#		0.221#	
Pearson Chi-square value	6.376		1.496	
8. Ondansetron with antimalarials or macrolides				
Increased	83.7 (169)	82.5 (52)	87.7 (192)	75.3 (55)
Unchanged	16.3 (33)	17.5 (11)	12.3 (27)	24.7 (18)
*P* value	0.924*		0.026#	
Pearson Chi-square value	12.787		7.310	

*p values were calculated using Fisher's exact test, ^#^*p* values were calculated using Pearson Chi-square test

## Discussion

This is the first study to survey the pharmacists in Yemen to evaluate the pattern of prescription and nonprescription drugs demanded during COVID-19.

In this study, more than one-third of the participants were diploma holders and undergraduate pharmacy students who were acting as pharmacists and dispensing drugs to customers. This observation is not uncommon in Yemen, where many studies have reported that unqualified people in pharmacies are playing the role of pharmacists. This is because of the shortages in pharmacists and the absence of authoritative actions against this act [13, 14]. Probably, the situation is prevailing in Yemen since the government, under the civil war, has other priorities than enforcing pharmacy practice regulations, and it does not have authority in some parts of the country.

In terms of drugs demanded, antibiotics were the highest prescription drugs in demand during the pandemic. This increase in antibiotic demand was seen in other countries where COVID-19 has impacted the pattern of antibiotic utilization. In Pakistan, pharmacists reported an increase in antimicrobial utilization during COVID-19 [27]. In other countries, broad-spectrum antibiotics were prescribed to COVID-19 patients co-infected with a bacterial infection [28–30]. The most prescribed antibiotics in these studies were fluoroquinolones and third-generation cephalosporins. In our study, cephalosporins comprised 47% of the demanded antibiotics (Table 2); however, no increase in fluoroquinolone demand was found. In Yemen, the problem of excessive administration of cephalosporins was reported before the pandemic [31]. Yemeni people are familiar with cephalosporins for the treatment of respiratory infections because it is one of the highly prescribed antibiotics for outpatients, comprising 39% of all prescriptions [32]. Since COVID-19 has respiratory symptoms, this could explain the increase in cephalosporin demand during the pandemic.

An increase in the demand for antimalarial drugs was also reported in this study (Table 2). Antimalarials are a familiar drug category among the Yemeni community due to the endemicity of malaria in the country [33, 34]. In addition, at the time of this study, there were several discussions in the media about the antimalarial drug "hydroxychloroquine" as a novel therapeutic agent in the treatment of COVID-19 [35]. At that time, hydroxychloroquine and azithromycin were recommended according to some observational studies and treatment protocols for COVID-19 patients [36, 37]. This information was picked up by social media and heavily circulated among the general population. Self-medication with hydroxychloroquine was documented during the COVID-19 pandemic in similar low-income countries such as Bangladesh and Peru [38, 39]. In Yemen, another reason alongside COVID-19 that could explain the increased demand for antimalarial drugs was the emergence of a malaria outbreak [40]. Malaria and dengue fever outbreaks were linked to a heavy flood that occurred in March and April 2020, providing an ideal environment for mosquito-borne diseases [41]. In general, people residing in similar endemic countries are familiar with antimalarials, and in Yemen, access to these drugs does not necessarily require a prescription, which might explain the emergence of chloroquine-resistant malaria a decade ago [33, 34]. The use of hydroxychloroquine or many other antimalarials has a potential risk on the heart by prolonging QT-interval [42]. Thus, self-medication with antimalarials without medical supervision might be life-threatening. Several cases of severe poisoning were reported in Nigeria due to self-medication by combining both hydroxychloroquine and azithromycin [43]. Therefore, Yemeni pharmacists should be well educated and cautious about the risks associated with this common practice of the public and responsible enough not to dispense these drugs to patients without a prescription.

Another serious issue identified in this study was that 74.8% of the participants indicated an increase in dispensing ondansetron's with antimalarials and antibiotics (Table 2). In a community acquainted with antimalarials, people are familiar with their gastrointestinal side effects, such as nausea and vomiting, which drive people to take anti-emetic drugs. However, among the side effects of ondansetron is its effect on the cardiovascular system, which includes QT-interval prolongation [44], an adverse drug reaction that is also reported with macrolides and quinolone antibiotics and hydroxychloroquine [42]. Co-administration of drugs with potential cardiac effects is a significant problem when both drugs prolong the QT-interval since this may increase ventricular arrhythmias and the risk of sudden death [45].

It is well known that pandemics are accompanied by psychological distress and anxiety due to lockdown conditions and intolerance to uncertainty about expected death from this disease [46]. Consequently, this mental health issue is expected to increase the demand for anxiolytic drugs, as reported in different countries. In the USA, the prescription for antianxiety drugs was increased to 34% in one month [47]. In France, Addictovigilance (a safety monitoring organization targeting substances with potential for abuse and dependence) has reported increased events of sedatives and anxiolytics overuse and abuse during the lockdown from March to May 2020 [48]. In our study, a substantial increase in the demand for sedatives and anxiolytics was reported by 70.6% of the participants. In Yemen, the problem of sedatives and anxiolytics abuse is increasing alarmingly as was declared earlier by Yemeni community pharmacists in 2013 [18], and unfortunately, no action has been taken to regulate the dispensing of such drugs due to the civil war ravaging the country.

During COVID-19 in Yemen, an increase in the demand for antithrombotic agents was identified by 67% of the participants. Aspirin and enoxaparin were the most demanded antithrombotics. Antithrombotic agents have been included in the management guidelines for COVID-19 [49, 50]. This finding was broadcast in national and international news and shared on social media. We believe that this information, which was picked up by the desperate public for the use of any medication to treat or prevent the severe outcomes of COVID-19 has resulted in the increased demand for antithrombotic drugs. This issue is alarming since the use of antithrombotic agents without medical indication may lead to bleeding. Furthermore, in a country struggling with endemics such as hemorrhagic dengue fever, the use of antithrombotics might lead to an uncontrollable crisis. It has been reported that the continuation of platelet aggregation inhibitors such as aspirin for heart failure patients who are infected with dengue fever might lead to fatal hemorrhage [51]. Dengue fever also has respiratory manifestations like COVID-19 [52], which can lead to misdiagnosis [53]; therefore, the administration of antithrombotics to these patients may complicate their disease outcome.

Regarding nonprescription drugs, paracetamol and vitamins were the highest demanded drugs/supplements during COVID-19 in Yemen (Table 2). An increase in the demand for analgesics/antipyretics is expected in COVID-19 since some of its most common symptoms are fever and muscle aches [54, 55].

On the other hand, the increase in the demand for codeine–paracetamol identified in this study should be worrying since this combination was reported as a drug of abuse in an earlier study in Yemen [18]. The abuse of this combination might worsen the condition of COVID-19 patients who suffer from respiratory complications given that codeine is known to have serious side effects, including respiratory depression [56].

In this study, the increase in the demand for vitamins was declared by 93% of the participants. This is expected since among the recommended home remedies for treating COVID-19 are vitamins to boost immunity [57–60]. This growth in demand for vitamins during COVID-19 was also seen worldwide [61, 62].

More than half of the participants (50.6%) indicated that the increased demand for prescription drugs was without providing any valid prescription during the pandemic in Yemen (Fig. 2). This practice was prevailing even before COVID-19 due to the absence of strict regulations for rational antimicrobials dispensing [63, 64]. In a study conducted in the district of Thamar in Yemen, about 82% of patients indicated that they had consumed antibiotics of different classes dispensed without prescription [63]. In another study, the dispensing of other prescription drugs, antidepressants, and anxiolytics without prescription was reported in Yemen before COVID-19. Some reports had shown an increase in the abuse and misuse of these drug categories [18]. Although there are some regulations about the dispensing of prescription drugs in Yemen, they are not enforced, partly because of the civil war which has almost paralyzed the authorities [13, 14]. Therefore, since enforcing the regulations cannot be achieved in the civil war situation, it is important to increase awareness campaigns targeting the public about the risks of using medications without proper diagnosis. These campaigns through different media should refute all the wrong beliefs and misinformation about the use of drugs for self-treatment and explain the dangers of this practice.

The demand for ondansetron with antimalarials or macrolides was significantly associated with major cities or unstable districts (*p* =0.026; Table 3). These districts are struggling with different multi-epidemics such as malaria, typhoid, cholera, dengue fever, and diphtheria [65] which explains the population's experience with antimicrobials side effects such as emesis. This in turn drives the patients to request anti-emetics. Therefore, pharmacists should be aware of possible severe antimicrobials–ondansetron interactions, as explained earlier.

The factors that could affect the change in the demand for drugs during COVID-19 were investigated. There was no statistical association between the increase in the demand for the majority of prescription and nonprescription drugs and the type of district (unstable or frontline district) or the type of pharmacy (community or hospital; Table 3). However, the increased demand for vitamins was significantly associated with community pharmacies more than hospital pharmacies (*p*-value = 0.012). Vitamins and supplements are over-the-counter drugs available in community pharmacies and can be easily procured from them. Accordingly, the patients would obtain these drugs from community pharmacies that are more accessible than hospital pharmacies, which could be closed due to hospitals' poor situations. Besides, patients could be cautious and prefer community pharmacies over hospital pharmacies as they fear contracting COVID-19 in hospital settings.

On the other hand, although most participants confirmed that they received updated information about the COVID-19 pandemic, the quality and accuracy of the received information are questionable since they depend on the source of this information. The major sources of information about COVID-19 for the participants were the Yemeni Ministry of Health, the Yemeni Pharmacists' syndicate, and the WHO-official website. Less than 2% of the participants used scientific journals as a source of information. According to Al-Jamei et al. (2019), the majority of Yemeni pharmacists were considered poor users of bibliographic databases for searching, where 74% of the study participants were unfamiliar with international guidelines: Cochrane, Medline, or PubMed databases [23]. We believe that in war zone countries, where government arms are not well functioning, civil organizations such as pharmacist syndicates or other healthcare syndicates should be supported by WHO or the United Nations (U.N.) to be able to disseminate knowledge and awareness among the pharmacists. Moreover, international agencies should consider, besides humanitarian aid, training and educating the workers in pharmacies to increase their knowledge and understanding about the risks of dispensing drugs without proper diagnosis. Also, in the case of pandemics, training is needed to educate pharmacists about the mild infection cases that can be dealt with without referring the patient to the physicians, to reduce the pressure on the physicians and ease the ways of treating patients to be able to confront the pandemic.

## Conclusion

The pandemic of COVID-19 in conflict and crisis countries such as Yemen worsened irrational drug use. The misuse and overuse of many prescription and non-prescription drugs have been reported in this study in addition to the increased demand for prescription drugs without providing a prescription. Undoubtedly, the ongoing war in Yemen negatively impacted healthcare services in general and extended the pharmacist's role to become the primary care provider and drug prescriber. Therefore, since the functioning of authorities in war times is adversely affected, policymakers and international agencies such as the U.N. or WHO should consider training and educating pharmacists significantly when outbreaks worsen the situation. In addition, it is very crucial to increase the awareness of the public about the risks of self-medication without knowing the proper diagnosis.

## Limitations of the study

There are some limitations in this study. The study was based on participants’ perception, which could be subjective and reflects the view of those who participated in the study. It was also based on the ability of the participants to recall information which could result in some inaccuracies in their responses. The study was based on a convenient sample size and limited to those who have access to the internet which limited the representation of all pharmacists.

## Supplementary Information

**Additional file 1.** Questionnaire/Online-survey.

## Data Availability

All data generated or analyzed during this study are included in this published article and its supplementary information files.
