# Supplementary material for: Changes in drug demand when a pandemic coincides with other outbreaks in a war zone country: a cross-sectional pilot study
Source: J Pharm Policy Pract. 2022 Nov 22;15:89. doi: 10.1186/s40545-022-00487-z (PMC9682693; doi:10.1186/s40545-022-00487-z)
Supplement: Supplementary file 1 — Additional file 1. Questionnaire/Online-survey. [file 40545_2022_487_MOESM1_ESM.docx]

**Supplement 1**

**Questionnaire/ Online-Survey**

Changes in the demand of Prescription and Non-prescription Drugs during the Pandemic of COVID-19: Pharmacists Perspectives in Yemen (March-May 2020)

**Information about the study**

This study is carried out by a team of researchers to measure changes in the pattern of drug demand during COVID-91 pandemic in Yemen by collecting information and observations by pharmacy workers.

Due to the closure of most outpatient clinics and some hospitals as a result of the pandemic, the pharmacy is the first resort for patients. We ask the pharmacist to help us in this questionnaire about what he/she observed regarding the pattern of drug demand during this health crisis.

This questionnaire is only intended for pharmacy workers who dispense or sell medicines in the pharmacies they are working in. Your participation is voluntary. You will not be asked to disclose your name or identity during the survey. This study aims at scientific research only. Completing and submitting the questionnaire signifies your agreement to participate in the study.

If you have any questions about this questionnaire, please contact *[ Dr Ebtesam Saleh*, via e-mail : ph.ebtisam@gmail.com ].

Thank you in advance for your cooperation.

| Section ADemographic data |  |
| --- | --- |
| 1. Gender: (please tick one) |  |
| Male | 🞏 |
| Female | 🞏 |
|  |  |
| 1. What is your academic level? (please tick one) |  |
| Undergraduate student | 🞏 |
| Diploma | 🞏 |
| Bachelor | 🞏 |
| 1. Postgraduate (MSc./PhD) | 🞏 |
| 1. Others | 🞏 |
|  |  |
| 1. The pharmacy school which you graduated from/still enrolled? |  |
| Private school in Yemen | 🞏 |
| Government school in Yemen | 🞏 |
| In Eastern Europe | 🞏 |
| In Western Europe or Northern America | 🞏 |
| In Russia | 🞏 |
| In Arabian Gulf countries | 🞏 |
| In Northern Africa | 🞏 |
| In Levant countries | 🞏 |
|  |  |
| 1. The district in which the pharmacy you currently work in is located: |  |
| Aden | 🞏 |
| Abyan | 🞏 |
| Hadramout | 🞏 |
| Al-Dhale | 🞏 |
| Al-Hodaida | 🞏 |
| Al-Jouf | 🞏 |
| Thamar | 🞏 |
| Hajja | 🞏 |
| Lahij | 🞏 |
| Ma’reb | 🞏 |
| Sana’a | 🞏 |
| Saada | 🞏 |
| Shabowa | 🞏 |
| Taiz | 🞏 |
| Other  --------------------------------------- | 🞏 |
|  |  |
| 1. What is the nature of the area in which the pharmacy you work in? (please tick one). |  |
| Rural | 🞏 |
| Semi-urban | 🞏 |
| Urban | 🞏 |
|  |  |
| 1. What is the nature of your role in the pharmacy? (you can tick more than one answer) |  |
| Pharmacy owner | 🞏 |
| Pharmacy employee (community pharmacy) | 🞏 |
| Pharmacy employee (hospital pharmacy) | 🞏 |
| Trainee in the pharmacy | 🞏 |
| Other  ----------------------------------- | 🞏 |
| 7. How many years of practical experience do you have working in pharmacies? (please tick one) |  |
| < 1 year experience | 🞏 |
| 1-5 years | 🞏 |
| 6-10 years | 🞏 |
| 11-15 years | 🞏 |
| 16-20 years | 🞏 |
| 21-25 years | 🞏 |
| More than 25 years | 🞏 |
|  |  |
| 8. During the past 6 months, how many training workshops in your field of work in the pharmacy (related to diseases, treatments, dealing with patients, dispensing medicines, etc.) have you attended, whether in-person or virtual? (please tick one) |  |
| 0 | 🞏 |
| 1 | 🞏 |
| 2 | 🞏 |
| 3 | 🞏 |
| 4 | 🞏 |
| 5 | 🞏 |
| > 5 | 🞏 |
|  |  |
| 9. Are you aware of and following up on the latest recommendations and treatment plans for Coronavirus (COVID-19)? |  |
| Yes | 🞏 |
| No | 🞏 |
|  |  |
| **If yes, please go to question 10. If no, please continue to Section B.** |  |
| 10. The source of your information about the latest developments in the treatment and control of the COVID-19, is? (you can choose more than one answer) |  |
| Awareness campaigns from the Pharmacists Syndicate | 🞏 |
| Awareness campaigns from the Ministry of Health | 🞏 |
| Individual-youth / community awareness campaigns | 🞏 |
| Newspapers or local newspapers | 🞏 |
| World Health Organization via its official website | 🞏 |
| Scientific electronic websites | 🞏 |
| Research that has been published in scientific journals | 🞏 |
| Some colleagues | 🞏 |
| Social media (WhatsApp, Facebook..etc) | 🞏 |
| Family and relatives | 🞏 |
| Others | 🞏 |
| ----------------------------------- |  |
|  |  |
| **Section B. Patterns of drug demand (Prescription and non-Prescription)**  The following questions aim to evaluate any increase in the demand of 6 classes of drugs in the last 3 months, March to May, 2020 from your perspective. |  |
| 11. During the past three months, has there been an increase in the demand for antimicrobial drugs (bacterial) by the residents of Yemen? If yes, choose the specific drug with increased demand, if no, choose "No, the demand of these drugs didn’t change" (You can choose more than one answer) |  |
| Macrolides such as (azithromycin, clarithromycin, etc) | 🞏 |
| Penicillins (amoxicillin, ampicillin, oxacillin, etc) | 🞏 |
| Oral Cephalosporins (Cephalexin, cefaclor, cefixime, cefuroxime, etc..) | 🞏 |
| Parenteral cephalosporins, (cefotaxime, ceftriaxone, cefepime, etc) | 🞏 |
| Fluoroquinolones (Ciprofloxacin, levofloxacin, moxifloxacin) | 🞏 |
| Aminoglycosides (gentamycin, amikacin, tobramycin) | 🞏 |
| No, the demand of these drugs didn’t change | 🞏 |
|  |  |
| 12. During the past three months, has there been an increase in the demand for antimalarial and or antiviral drugs by the residents of Yemen? If yes, choose the specific drug with increased demand, if no, choose "No, the demand of these drugs didn’t change" (You can choose more than one answer) |  |
|  |  |
| Antimalarial ( Hydroxychloroquine, Chloroquine, Artemether..etc) | 🞏 |
| Acyclovir | 🞏 |
| Oseltamivir | 🞏 |
| Amantadine | 🞏 |
| No, the demand of these drugs didn’t change | 🞏 |
|  |  |
| 13. Have you noticed an increase in the demand to dispense the nausea prevention medicine Ondansetron with one of the following medicines: Antimalarials, Fluroquinolones or Macrolides |  |
| Yes | 🞏 |
| No | 🞏 |
|  |  |
| 14. During the past three months, has there been an increase in the demand for analgesics and antipyretics by the residents of Yemen? If yes, choose the drug with increased demand, if no, choose "No, the demand of these drugs didn’t change" (You can choose more than one answer) |  |
| Oral paracetamol | 🞏 |
| Parenteral paracetamol | 🞏 |
| Oral ibuprofen | 🞏 |
| Oral meloxicam | 🞏 |
| Parenteral meloxicam | 🞏 |
| Oral tramadol | 🞏 |
| Parenteral tramadol | 🞏 |
| Codeine combinations | 🞏 |
| Oral ibuprofen | 🞏 |
| No, the demand of these drugs didn’t change | 🞏 |
|  |  |
| 15. During the past three months, has there been an increase in the demand for antithrombotic by the residents of Yemen? If yes, choose the drug with increased demand, if no, choose "No, the demand of these drugs didn’t change" (You can choose more than one answer) |  |
| Aspirin | 🞏 |
| Clopidogrel | 🞏 |
| Clexane | 🞏 |
| Heparin | 🞏 |
| Warfarin | 🞏 |
| No, the demand of these drugs didn’t change | 🞏 |
|  |  |
| 16. During the past three months, has there been an increase in the demand for anxiolytics and sedatives by the residents of Yemen? If yes, choose the drug with increased demand, if no, choose "No, the demand of these drugs didn’t change" (You can choose more than one answer) |  |
| Benzodiazepines (Diazepam, Lorazepam, Clonazepam ...etc) | 🞏 |
| Barbiturate (Barbital, Phenobarbital…etc.) | 🞏 |
| Opioids (Oxycodone, Fentanyl, Methadone....etc) | 🞏 |
| Antihistamines (Chlorpheniramine, Cyclizine, Orphenadrine…etc) | 🞏 |
| Antidepressants (sertraline, citalopram, fluoxetine, ...etc) | 🞏 |
| Pregabalin | 🞏 |
| No, the demand of these drugs didn’t change | 🞏 |
|  |  |
| 17. During the past three months, has there been an increase in the demand for vitamins, minerals and supplements by the residents of Yemen? |  |
| Yes | 🞏 |
| No | 🞏 |
|  |  |
| 18. Please add any other medicines that were not mentioned above, and which you notice an increase in the demand for in the last three months.  *Thank you for your time and valuable answers.* |  |
|  |  |
